# From behavioral signals to therapy management: a real-world pilot implementation of human-in-the-loop AI workflow in proactive patient care

**DOI:** 10.3389/fdgth.2026.1887889

**Published:** 2026-07-27

**Authors:** David Dickerson, Koeun Lim, Caitlin Tourjé, Ramo Naidu, Lindsey Cianni, Andrew B. Kibler

**Affiliations:** 1 Endeavor Health, Chicago, IL, United States; 2 Pritzker School of Medicine, University of Chicago, Chicago, IL, United States; 3 BIOTRONIK NRO Inc., Lake Oswego, OR, United States; 4 Spanish Hills Interventional P&S, Camarillo, CA, United States; 5 MarinHealth Spine Ins., Larkspur, CA, United States

**Keywords:** artificial intelligence, digital health implementation, human-in-the-loop AI, neuromodulation, patient care decision support, patient care workflow integration, post-implant care, remote monitoring

## Abstract

**Background:**

Spinal cord stimulation (SCS) is an established therapy for chronic pain, but post-implant care depends on timely identification of evolving patient needs. Remote monitoring may help detect therapy-use changes that scheduled follow-up and patient-initiated contact can miss. We evaluated Proactive Intelligence, a workflow-integrated, human-in-the-loop AI recommender using passively collected SCS device-interaction data to support care without added patients’ data-entry burden.

**Methods:**

We conducted a prospective, observational real-world pilot among research-consented patients implanted with Prospera SCS devices. Proactive Intelligence used a hybrid neural network incorporating longitudinal therapy-use patterns, operational variables, and patient history/demography to identify patient-days with therapy-adjustment-associated patterns. Model outputs were translated into a prioritized daily review list for the Embrace Care Team (ECT), which reviewed cases, performed outreach when appropriate, adjudicated behavioral-change reasons, and recorded downstream patient care actions. Outcomes included model enrichment, field adjudication yield, intervention distribution, and pain-score change after outreach when follow-up pain scores were available.

**Results:**

Model development used data from 747 permanently implanted patients with sufficient telemetry. Confirmed reprogramming events were rare, occurring at 0.77% per patient-day. At the high-specificity threshold, the model achieved a positive predictive value of 28.8% and sensitivity of 8.6% on a held-out test set, corresponding to approximately 37-fold enrichment over baseline prevalence. During the pilot, predictions were generated across 101,042 patient-days. Across 91 operational days, the ECT reviewed 188 cases involving 175 unique patients. Among reviewed cases, 152 patients responded within three outreach attempts, yielding an 80.9% response rate. Of all reviewed cases, 66.0% were adjudicated as “therapy-related.” Among reachable patients, the therapy-related yield was 81.6%, and “Address therapy” actions occurred in 67.8% of cases, including reprogramming and therapy adjustment/discussion. Among cases with follow-up pain scores, therapy-relevant cases showed significant mean NRS reduction after outreach, with improvement after both reprogramming and non-reprogramming therapy adjustment.

**Discussion:**

To our knowledge, this pilot represents the first real-world evaluation of an ECT driven, human-in-the-loop care workflow supported by passively collected SCS device interaction data, without added patient data-entry burden. The findings suggest that machine learning can assist longitudinal SCS care by surfacing behaviorally meaningful changes for timely human review, contextual interpretation, and coordinated support.

## 1 Introduction

Spinal cord stimulation (SCS) is an established therapy option for appropriately selected patients with chronic back and lower extremity pain, with previous randomized control trials and observational studies showing superior improvements versus conventional medical management for multiple patient outcomes at 6 months and beyond (1–4). In routine practice, however, SCS effectiveness is not established solely at implantation or at a single post-implant time point. Post-implant outcomes may depend on multiple factors, including sustained engagement with therapy and timely identification of evolving needs, such as optimization of stimulation delivery or resolution of practical barriers that prevent effective use.

After implantation, therapy management is inherently iterative. Specifically, modern systems involve multiple programmable dimensions (e.g., contact selection, amplitude, pulse width, frequency), and different combinations are configured as “programs” to support patient-specific responses and activity-dependent needs (5). Consensus guidance in neuromodulation further emphasizes that postoperative care, such as optimization, troubleshooting, and salvage strategies when outcomes are suboptimal, should be approached systematically, with the goal of improving sustainable benefit and avoiding preventable loss of therapy before considering explant (6).

Despite this clinical reality, post-implant SCS care is often constrained by workflows that may limit timely detection of emerging issues between scheduled touchpoints. Expert recommendations on remote SCS device management including remote monitoring and remote programming frame these tools as an opportunity to reduce burdens of in-person-only workflows while supporting prompt identification and resolution of SCS-related issues (7, 8). Importantly, these recommendations clarify that remote monitoring can involve a broad set of metrics including device-related measures such as stimulation usage, along with physiological proxies and patient-reported measures. Furthermore, it was recommended that periodic review be tailored to individual needs rather than applied as a one-size-fits-all process (8). However, relying on scheduled visits or patient-initiated check-ins can leave meaningful changes in patient state undetected between interactions, while attempts to close this gap through repeated blind outreach would increase patient burden and intrusiveness. Advanced signal monitoring may help address this “surveillance gap” by passively capturing behavioral changes that can support timely intervention or escalation.

A related, underappreciated gap is that lack of follow-up is not synonymous with success. In a cross-sectional phone survey study of patients deemed lost to in-person follow-up after SCS implantation, up to 19% were quickly lost to follow-up at 1-month after being implanted (9). Among reachable patients, reasons spanned improvement in pain, minimal improvement, relocation, noncompliance with follow-up, and device-related barriers such as difficulty recharging (9). This heterogeneity illustrates a practical challenge for post-implant care. In other words, the same observable behavioral signal—e.g., reduced engagement with therapy or clinic interactions—can map to very different underlying causes that can be either favorable, neutral, or requiring intervention. To triage and interpret these signals, scalable processes are needed. This problem is amplified by a fundamental limitation in clinical AI development: label scarcity (10, 11). In many real-world healthcare data, the clearest “positive” labels arise only when patients actively initiate contact or otherwise generate a documented care event. As a result, the overwhelming majority of patient-days remain “unlabeled” rather than confirmed “negative” (10, 11). This creates an important distinction from traditional supervised machine learning (ML) where a “false positive” is typically treated as a model error. Such conventional interpretation can be misleading in label-scarce, post-implant SCS care workflows. Because “non-positive” cases are frequently unlabeled rather than truly negative, a flagged patient who does not proceed to a therapy reprogramming is not necessarily a model failure. The case may involve a patient whose behavior changes reflect: (a) improvement and reduced need for support; (b) temporary life or access barriers; (c) adherence, charging, education issues; or (d) a need for proactive check-in that can be resolved without clinician intervention. The loss-to-follow-up literature underscores that disengagement can reflect both improvement and unmet needs, including device-related barriers (9). Similarly, remote management guidance emphasizes that monitoring metrics including stimulation usage are useful precisely because they can prompt timely identification and resolution of issues, but interpretation must remain individualized and behaviorally contextual (8).

To address these unmet needs in post-implant patient care, we developed a hybrid neural network (HNN) model operationalized as a model-assisted recommender rather than an autonomous decision-maker. The model intakes daily, passively collected system usage data which is transmitted as part of remote monitoring. The end user is the Embrace Care Team (ECT), who interacts directly with patients to support SCS therapy use. Proactive Intelligence generates a prioritized list of patients whose therapy-usage behavior shows changes resembling previously observed high-signal exemplars. The ECT then reviews available patient information including therapy usage patterns and relevant context and decides whether to reach out to the patient, monitor without outreach, or take other non-clinical support actions such as automated escalation pathways when appropriate. This design is intentionally human-in-the-loop and explicitly avoids providing clinical advice. It aligns with national and international standards for AI development in healthcare. This positioning is consistent with broader ethical and adoption-oriented framing that AI in medicine should augment, not replace clinical care processes, and that trust and acceptance are strengthened when systems preserve meaningful human oversight (12).

Here we report the real-world pilot of Proactive Intelligence as a workflow-integrated recommender for the ECT, focusing on field performance and real-world impact such as triage utility, operational learnings, and patient-support actions rather than benchmark-style accuracy on large labeled test sets. In this pilot, cases that would conventionally be labeled as “false positive” were operationally informative. These cases suggested that the model often detecting the same behavioral signature arising from different underlying reasons, allowing us to refine both the model and the downstream operational playbook rather than dismiss these detections as simple errors. We emphasize that real-world deployment provided an essential validation layer for understanding what the model surfaced in practice and for iteratively improving model behavior and operational protocols.

## 2 Materials and methods

**Figure 1:**
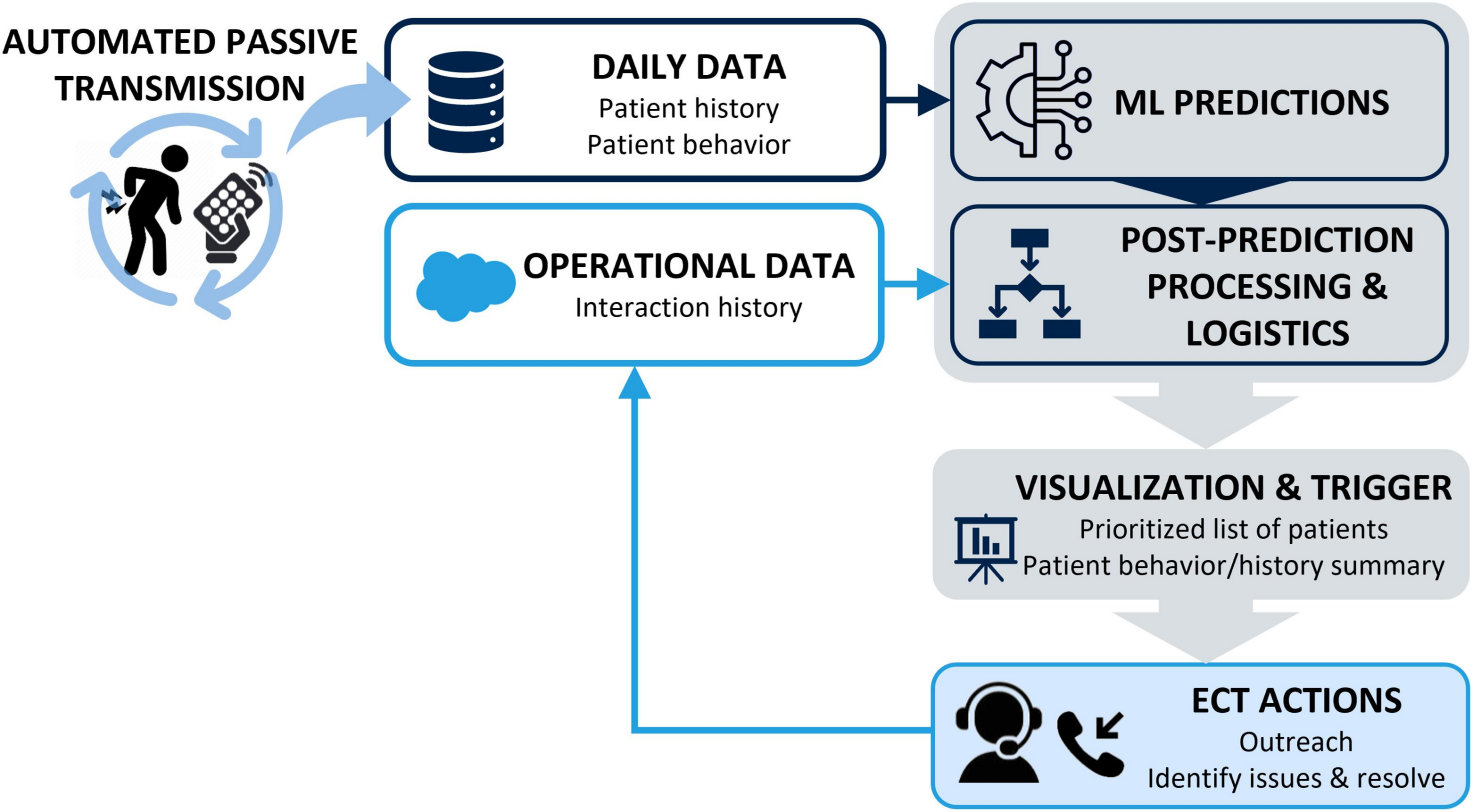
Human-in-the-loop AI integration workflow. Daily therapy-use behavior and patient history data are used to generate machine learning predictions, which are further refined through post-prediction processing and logistics to produce a prioritized review list with patient behavior and history summaries. This information supports targeted ECT outreach, issue identification, and resolution within a human-in-the-loop operational workflow.

We conducted a prospective, observational real-world pilot evaluation of Proactive Intelligence, a human-in-the-loop patient care operations tool intended to support post-implant SCS care by identifying patients showing behavioral changes in therapy usage combined with other historical and demographical traits that may warrant ECT review as illustrated in Figure 1. The pilot was designed as an implementation and feasibility study focused on field actionability and workflow impact rather than offline benchmark accuracy. Proactive Intelligence does not generate clinical recommendations or programming settings and does not direct physician decision-making. It provides a prioritized signal for the ECT to review patient information and decide whether to reach out or take other support actions.

### 2.1 Participants

The pilot participants consisted of patients who were permanently implanted with Prospera SCS devices. Inclusion criteria for the Proactive Intelligence pilot were: (a) consented to research and remote monitoring; (b) permanently implanted for more than 90 days; and (c) active and recent device use with available utilization data. Exclusion criteria were: (a) insufficient device utilization data to compute change features; and (b) administrative constraints such as opt-outs preventing outreach. The model development cohort and pilot-eligible population were distinct by design. The model development cohort represented a fixed historical dataset of patients who met model development criteria at the time the training and holdout datasets were assembled. In contrast, the pilot-eligible population represented a dynamic daily operational cohort during prospective deployment. On each prediction day, patients were included if they were permanently implanted, met the required post-implant duration criterion, had applicable research and remote-monitoring consent, and had sufficient recent device interaction data to construct the model input window. As additional patients became newly implanted, matured beyond the post-implant eligibility threshold, consented to remote monitoring/research use, or accumulated sufficient telemetry, they could enter the eligible pool.

### 2.2 Machine learning

**Figure 2:**
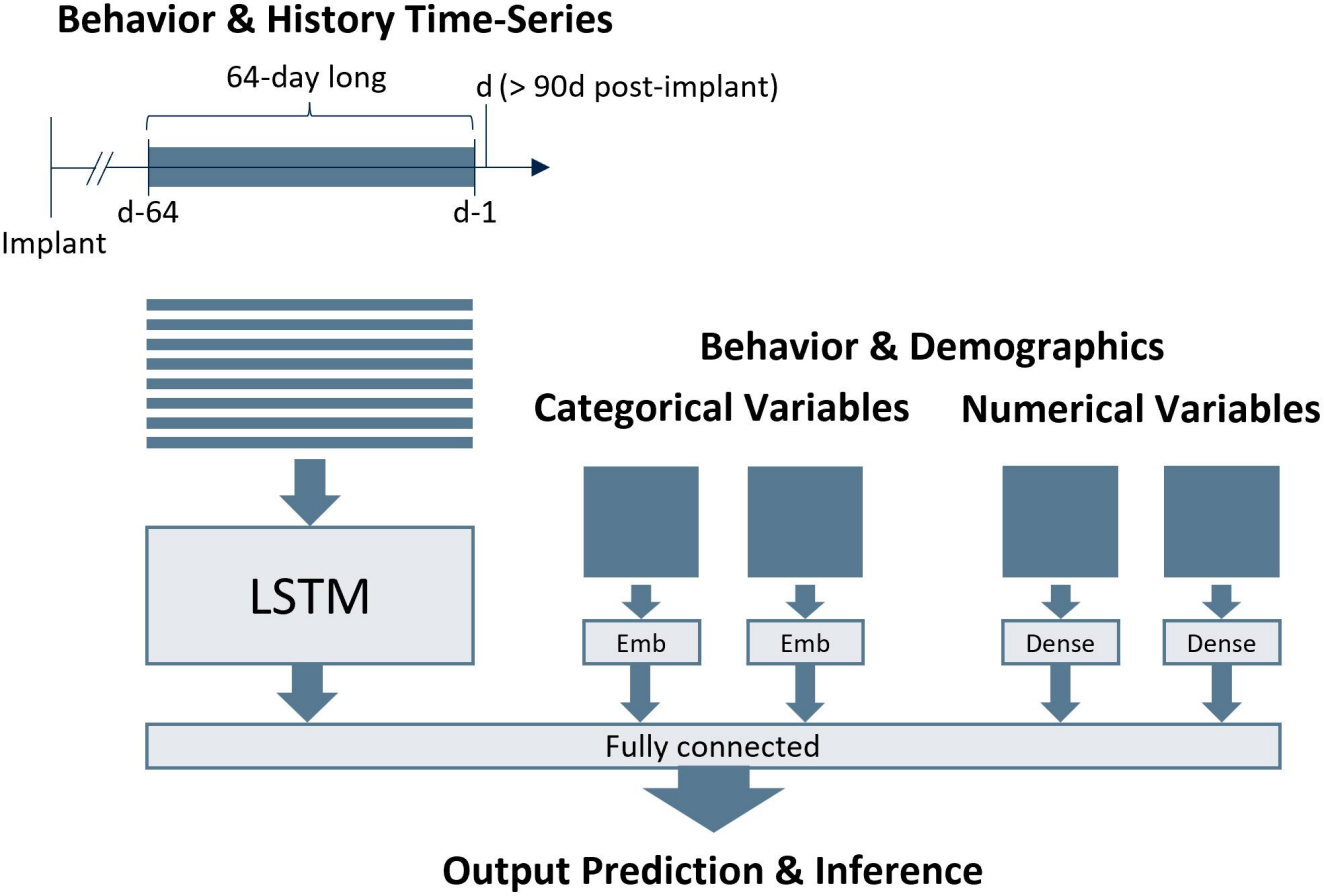
Hybrid neural network (HNN) architecture.

Proactive Intelligence is an HNN classifier that combines long short-term memory (LSTM) (13) with embedding and dense layers to handle time-series, categorical, and numerical input features as illustrated in Figure 2. The hybrid LSTM architecture was selected as a pragmatic multimodal structure for heterogeneous operational inputs. The time-series inputs reflecting patient behavior/history were passed through a single-layer LSTM with 128 hidden units. Categorical variables were processed using separate embedding layers, and numerical inputs were processed using a fully connected layer with ReLU activation. No separate validation set or formal hyperparameter search was used. The model was trained using fixed prespecified parameters: Adam optimizer, learning rate of 0.001, batch size of 32, 25 training epochs, and binary cross-entropy loss. Embedding dimensions were 9 for geographical region, 1 for gender, and 5 for primary indication; age was processed through a fully connected layer with 4 hidden units. A sliding-window approach was not implemented because the dataset available at the time of model development was limited. Instead, the model used the available longitudinal data for each patient. The final architecture was intentionally shallow, which was selected after exploration of deeper architectures did not show sufficient benefit to justify the added model complexity. No other explicit regularization was applied. The architecture was selected to support prospective workflow deployment rather than through formal comparative benchmarking.

The model outputs a probability score and corresponding binary prediction (0 or 1) indicating whether a patient-day exhibits a behavioral change pattern consistent with previously observed usage trajectories associated with therapy adjustment. The model is intentionally optimized for sensitivity to rare, high-signal behavioral patterns under label scarcity. In other words, offline discrimination metrics were considered supportive development diagnostics rather than definitive evidence of clinical utility.

#### 2.2.1 Data

Model inputs were derived from daily therapy usage data automatically transmitted within BIOTRONIK Neuro Remote Monitoring infrastructure, thereby minimizing patient burden for data collection. Only the data from research-consented patients were used. The analytic unit for model inference was a patient-day, enabling the model to continuously assess changes in therapy utilization behavior relative to each patient's recent history. Input data consisted of average therapy amplitude, therapy type, program/amplitude change counts, stimulus off, patient indication, gender, age, follow-up history, and geographical region. For each patient-day evaluated, a fixed-length longitudinal usage history was constructed to represent behavioral dynamics. Follow-up history and geographical region were included based on care team domain knowledge and iterative workflow development. These variables were considered relevant to interpreting therapy-use behavior in a real-world SCS care workflow because device interaction patterns may be influenced by recent support history, regional support patterns, patient education, field-team interaction, and access to care resources.

#### 2.2.2 Preprocessing and missing-data handling

For each scored patient-day, we constructed multiple 64-day utilization time-series representing therapy usage behavior leading up to that day. Behavioral change events of interest were defined operationally as changes occurring within this 64-day window, such that the model could learn and detect deviations in usage patterns over a clinically relevant, near-term horizon. The 64-day horizon was selected to balance sensitivity of detecting recent dynamics with robustness to short-lived noise and intermittent data gaps. Patients were included if they had fewer than 26 missing days within the 64-day window and fewer than 3 missing days within the most recent 7. These criteria were applied before model prediction to reduce scoring based on sparse or unreliable telemetry. Missing values within eligible input windows were imputed using (i) forward fill for average amplitude and therapy type and (ii) median for program/amplitude change and stimulus off counts. For categorical variables, missing values were imputed with “Unknown”, and missing patient age values were imputed using the cohort mean age.

#### 2.2.3 Label definition: positive-unlabeled (PU)

Ground-truth “positive” labels were assigned when patients initiated contact and subsequently underwent a formal reprogramming procedure. Because these events were rare (<1% of patients per day) and because absence of reprogramming does not establish absence of need, non-positive cases were treated as unlabeled rather than true negatives. Accordingly, model development and interpretation followed a positive-unlabeled (PU) framing. Specifically, positive labels were assigned to patient-days associated with confirmed therapy-adjustment interactions whereas unlabeled patient-days were all other days without a confirmed reprogramming session. This framing informed both evaluation and interpretation of apparent “false positives,” which were expected to include behaviorally meaningful changes with heterogeneous underlying reasons.

#### 2.2.4 Data splitting

To reflect deployment conditions and reduce information leakage across time, data were split chronologically into development and evaluation segments (temporal split). Data through February 2025 were included, with February 2025 reserved as the hold out set. This design ensured that model training used only earlier data and evaluation used later data. This development-evaluation split targeted 80-20 split. Then within the development segment, data was randomly split 80-20, with 80% used for model fitting and 20% used as a development test set for discrimination evaluation. To address data imbalance, data sets associated with positive labels were oversampled in the training set. As the pilot continued, the model was retrained periodically every 2 weeks to mitigate data drift that occurred in real-world environments.

#### 2.2.5 Model training: exemplar-preserving (memorization-oriented) fitting under label scarcity

Given the rarity of confirmed positives and PU setting, training used a high-capacity fitting paradigm intended to preserve and amplify sparse, high-signal positive patterns. The model was trained beyond the point typically favored for generalized classifiers, such that development test AUC-ROC approached ∼0.99. We treated this behavior as consistent with an exemplar-preserving strategy in which the model learns sharp decision boundaries around rare, clinically meaningful signatures. Because non-positive cases were largely unlabeled, we did not interpret near-perfect offline AUC as a claim of broad generalization. Instead, field deployment outcomes were used as the primary measure of real-world value.

#### 2.2.6 Post-prediction processing: operational prioritization

Model outputs were translated into a daily workflow using post-prediction processing to enable patient prioritization with high confidence for review. Specifically, the model classification threshold was selected through threshold-sensitivity analysis and operational calibration rather than by applying a default cutoff of 0.5. Candidate thresholds were evaluated according to detection rate and positive predictive value. The threshold of 0.999 was selected as a first-stage high-specificity filter because it preserved an operationally meaningful detection rate while substantially reducing the candidate pool for review. Because the number of detected patients could still exceed the allocated workload, the deployed workflow used second-stage operational prioritization steps: (a) patients with a prediction threshold over 0.999 within the last 7 days were considered eligible for review; (b) then eligible patients were prioritized using detection recency and persistence. Operationally, this persistent behavioral change was prioritized over single-day anomalies. Each day, the ECT reviewed the highest-priority patients, targeting 2 patients per day.

### 2.3 Embrace care team human-in-the-loop workflow

The end user of Proactive Intelligence was the ECT, whose members interact with patients to support therapy use after SCS implantation. Figure 1 summarizes the operational workflow. The operational workflow consisted of: (i) daily review of the prioritized patient list produced by the post-prediction processing pipeline; (ii) case review using available patient information in routine support workflows (e.g., therapy usage and interaction history); which subsequently led to a (iii) patient care decision. The decision was to take one of the following actions: (i) reach out to the patient for supportive check-in including reprogramming and therapy adjustment/discussion, education, troubleshooting, or barrier identification; or (ii) monitor without outreach. Up to three outreach attempts were typically made using SMS and voicemail. When reaching out to patients, the numerical rating scale (NRS) of pain intensity between 0 (no pain) and 10 (most extreme pain) was collected when possible. If needed, patients were followed up after 7 to 30 days from the initial outreach and additional NRS scores were collected. The model did not provide programming settings, medical advice, or recommendations intended to direct physician practice. All clinical interpretations and decisions outside the ECT's scope remained with the patient's following physician.

### 2.4 Statistics

Descriptive statistics were used to summarize cohort characteristics, model development data, pilot operational throughput, field adjudication outcomes, and intervention outcomes. Continuous variables are presented as median (minimum to maximum), and categorical variables are presented as counts and percentages.

95% confidence intervals (95%CI) for the positive predictive value (PPV), sensitivity, and number needed to review (NNR) were calculated by estimating the Wilson score 95% confidence interval for the corresponding outcome proportion and then inverting the confidence limits. Differences in pain intensity at the time of outreach across field adjudication categories were evaluated using a linear fixed-effects model, with field adjudication category specified as a fixed effect. When assessing percent NRS reduction (pNRSr) after outreach, within each analysis group, one-sample, one-tailed *t*-tests were used to assess whether mean pNRSr was greater than 0, corresponding to improvement in pain after outreach or intervention. Where multiple comparisons were performed, *p*-values were adjusted using the Bonferroni correction. Statistical significance was defined as *p* < 0.05. Analyses were performed using MATLAB.

## 3 Results

### 3.1 Model development

Data from 747 patients who were permanently implanted and actively using SCS therapy for more than 90 days as of February 28, 2025 were used to develop an HNN model. Only patients with sufficient therapy usage telemetry were included. Within this cohort, the baseline prevalence of reprogramming sessions was 0.77% per day, corresponding to approximately 2.83 sessions per patient-year. To reduce temporal leakage and approximate deployment conditions, data was temporally split, resulting in 55,184 instances for development segment and 15,001 holdout instances (21%) for evaluation segment. Development segment was then randomly split 80-20 for training and testing. The HNN model achieved an AUC-ROC of 0.9954 on this randomly split test set, which was interpreted as a development-stage discrimination diagnostic rather than evidence of optimized generalizable performance. After each biweekly retraining cycle, train-set AUC-ROC was monitored as an internal quality-control measure to confirm that the retrained model continued to preserve the input patterns associated with positive labels. Across retraining cycles, train-set AUC-ROC ranged between 0.9884 and 0.9928 with a median of 0.9912.

On the temporally held-out dataset, at the selected operating threshold of 0.999, the model achieved a positive predictive value (PPV) of 28.8% [95%CI 24.0, 34.1%] and a sensitivity of 8.6% [95% CI 7.0, 10.5%]. Relative to the baseline event prevalence, this corresponds to an approximately 37-fold enrichment, indicating substantial concentration of true positive cases among flagged patients. In practical terms, this equates to approximately one true positive for every 3–4 flagged cases.

Although sensitivity at this operating point was limited, the threshold was selected to prioritize precision and maintain a manageable false-positive burden in a low-prevalence setting. This trade-off enables identification of a highly enriched subset of patients for targeted review.

### 3.2 Model interpretability

Model interpretability was assessed using SHAP (14) (SHapley Additive exPlanations)-based global feature importance analyses across the training dataset, the temporally held-out dataset, and the reviewed-case dataset (see Supplementary Figures S1–S3). Across these analyses, the most influential features were predominantly related to longitudinal therapy-usage behavior, device interaction patterns, and prior support history, with demographic variables contributing to a lesser extent.

Feature importance rankings were broadly consistent between the training and temporally held-out datasets, supporting that the model relied on stable signal patterns rather than highly dataset-specific artifacts. The reviewed-case feature importance profile also showed substantial overlap with these core predictors, while reflecting the feature patterns most relevant in the subset of cases that progressed through operational review. In particular, the dominant features across analyses included follow-up event history, stimulation toggle count, program change count, amplitude change count, stimulation-off ratio, waveform composition metrics, and selected contextual variables such as indication and geographical region.

Because many of the model inputs were temporal or aggregated features, interpretation of signed SHAP values at the individual-feature level was less straightforward. Individual feature contributions likely reflected interactions across time and across correlated usage patterns rather than isolated directional effects. Accordingly, interpretation focused on global importance and cross-dataset consistency rather than directional attribution of individual predictors.

In summary, these findings support that the model's predictions were driven by coherent and clinically plausible patterns in longitudinal therapy utilization data. The addition of reviewed-case feature importance further suggests that the cases reaching operational review were influenced by the same general feature families identified during model development, providing an additional layer of field relevance.

### 3.3 Real-world pilot operations

During the pilot period from August 2025 through March 2026, Proactive Intelligence generated daily predictions for research-consented SCS patients with sufficient daily therapy utilization data to construct the required input dataset. The number of patients who were eligible for generating predictions ranged between 1,149 and 1,915 (median 1,541). At the operational flagging threshold of 0.999, the model flagged 2.16% of eligible patients per day. Across the pilot period, 833 unique patients were flagged at least once by the model. After post-prediction processing (Figure 2) that incorporated recent operational history and persistence of flagging, the daily operational review pool was reduced to 1.89% of eligible patients per day.

Because the pilot study design operated under a targeted review capacity of 2 patients per operational day, only a selective subset of flagged cases proceeded to manual review. Over 91 operational days, the model generated predictions across 101,042 patient-days. After thresholding and prioritization, the ECT reviewed 188 cases corresponding to 175 unique patients. Table 1 summarizes the demographics of the overall eligible cohort, the model-flagged cohort, and the reviewed cohort as of March 31, 2026. Overall, the demographic profile of flagged patients was similar to that of the broader eligible cohort, although reviewed patients tended to be slightly older and showed a somewhat higher representation of complex regional pain syndrome (CRPS) and degenerative disc disease.

**Table 1:** Patient demographics as of March 31, 2026.

Charateristic	Overall Cohort	Model Flagged	Reviewed
Gender	%	%	%
Female	58.6%	58.6%	51.1%
Male	41.1%	40.9%	48.3%
Other	0.3%	0.5%	0.6%
Age, years	69 (22 to 97)	70 (23 to 96)	72 (34 to 91)
Time Since Implant, days	405 (91 to 1,072)	350 (92 to 1,072)	369 (102 to 985)
Primary Indication	%	%	%
Persistent Spinal Pain Syndrome (PSPS) Type II	51.2%	51.5%	43.0%
Radicular Pain Syndrome	24.6%	20.8%	19.4%
Degenerative Disk Disease	10.0%	10.6%	15.2%
Complex Regional Pain Syndrome (CRPS) Type I & II	8.5%	11.1%	15.7%
Other	5.7%	7.0%	6.7%

These results indicate that the model could be integrated into a targeted capacity workflow and maintain a target review volume. Importantly, the operational workflow was not designed to review every flagged patient. Rather, it was designed to prioritize the subset of flagged patients most likely to benefit from timely supportive follow-up.

### 3.4 Field adjudication outcomes: interpretation of flagged cases in practice

Because non-positive cases in this setting were unlabeled rather than true negatives, field adjudication was used as the primary mechanism to understand what the model was detecting in real-world practice. Among 188 reviewed cases (N), outcomes were categorized according to the predefined operational rubric shown in Table 2. Adjudication results are reported in Table 3.

**Table 2:** Field adjudication categories, reason codes, and operational descriptions.

Adjudication Category	Operational Description	Reason Code
A. Therapy-related	Patient report indicates insufficient benefit or desire for improved relief	R1. Seeking increased relief R2. New/worsened pain distribution R3. Unwanted stimulation effects (paresthesia/motor artifacts) R4. Disengagement
B. Behaviorally meaningful—therapy stabilization/routine	Patient reports benefit stabilization or intentional change in usage pattern	R5. Benefit stabilization R6. Intentional routine
C. Externally driven usage disruption	Externally driven usage disruption is identified—e.g., usage change due to travel/family/life/ health/usability/technical disruptions affecting access	R7. Life event (without change in pain state) R8. Competing health event R9. User error R10. Technical issues
D. Unable to adjudicate	Patients are unreachable after predefined attempts	R11. Unreachable

**Table 3:** Field adjudication results.

Adjudication	*n*	% of Reviewed Cases (*N* = 188)	% of Reachable Cases (*N*_re_ = 152)
A. Therapy-related	124	66.0%	81.6%
R1. Seeking increased relief	64	34.0%	42.1%
R2. New/worsened pain distribution	47	25.0%	30.9%
R3. Unwanted stimulation effects	10	5.3%	6.6%
R4. Disengagement	3	1.6%	1.9%
B. Therapy stabilization/routine	21	11.2%	13.8%
R5. Benefit stabilization	14	7.4%	9.2%
R6. Intentional routine	7	3.7%	4.6%
C. External disruption	7	3.7%	4.6%
R7. Life event	0	0.0%	0.0%
R8. Competing health event	2	1.1%	1.3%
R9. User error	4	2.1%	2.6%
R10. Technical issues	1	0.5%	0.7%
D. Unable to adjudicate	36	19.1%	–
Total Reachable	152	80.9%	100%

**Figure 3:**
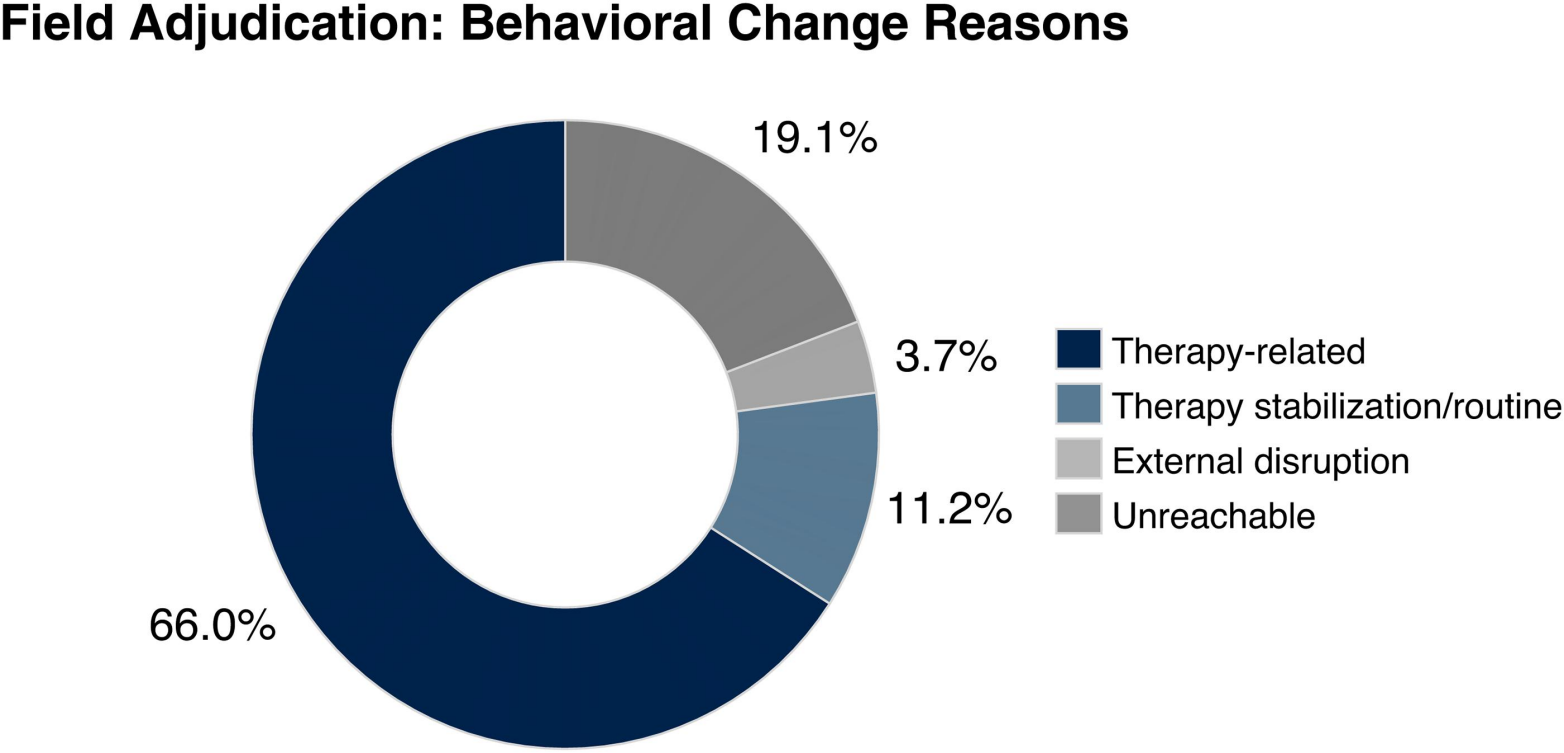
Distribution of field adjudication outcomes among reviewed cases. Categories are mutually exclusive and reflect the primary reason identified during ECT outreach and review.

Figure 3 shows the distribution of the field adjudication outcomes. Of the 188 reviewed cases, 152 patients responded within 3 outreach attempts, corresponding to a response rate of 80.9%, while 36 cases (19.1%) remained unreachable. Median case duration was 3 days, with lower and upper quartiles of 1 and 7 days, respectively. Among all reviewed cases, 124 (66.0%) were adjudicated as therapy-related (Category A in Tables 2, 3), indicating that the patient's reported experience was consistent with seeking increased relief (34.0%), new or worsened pain distribution (25.0%), unwanted stimulation effects (paresthesia/motor artifacts, 5.3%), or disengagement from therapy (1.6%). An additional 21 cases (11.2%) were classified as behaviorally meaningful but reflective of therapy stabilization or routine (Category B in Tables 2, 3). Seven cases (3.7%) reflected externally driven usage disruption (Category C in Tables 2, 3), such as travel, competing health issues, user error, or technical barriers.

Restricting analysis to reachable patients, 152 cases were reviewed eligible cases (N_re). Using Category A as the primary end point, the therapy-related yield was 81.6% ($n_{A}/N_{\mathrm{re}}\times 100$). This finding is operationally important because it indicates that most reviewed cases did not represent random noise. Rather, they reflected interpretable behavioral changes that were relevant to patient support, whether through active therapy optimization or through clarification of routine, stable, or intentional changes in therapy use.

Then, operational efficiency was quantified using NNR. Based on reachable cases, the NNR to identify one therapy-related case was 1.23 [95%CI 1.15, 1.34] ($N_{\mathrm{re}}/n_{A}$), and the NNR to identify one meaningful/support case was 1.05 [95%CI 1.02, 1.10] ($N_{\mathrm{re}}/(n_{A}+n_{B})$). When calculated using all reviewed cases, including unreachable patients, the NNR was 1.51 [95%CI 1.38 1.70] ($N/n_{A}$) for therapy-related cases and 1.30 [95%CI 1.21, 1.42] ($N/(n_{A}+n_{B})$) for meaningful/support cases. These values indicate substantial enrichment of practically useful cases within the reviewed pool, especially given the low baseline prevalence of confirmed reprogramming events in the underlying population.

### 3.5 Pain intensity at outreach

**Figure 4:**
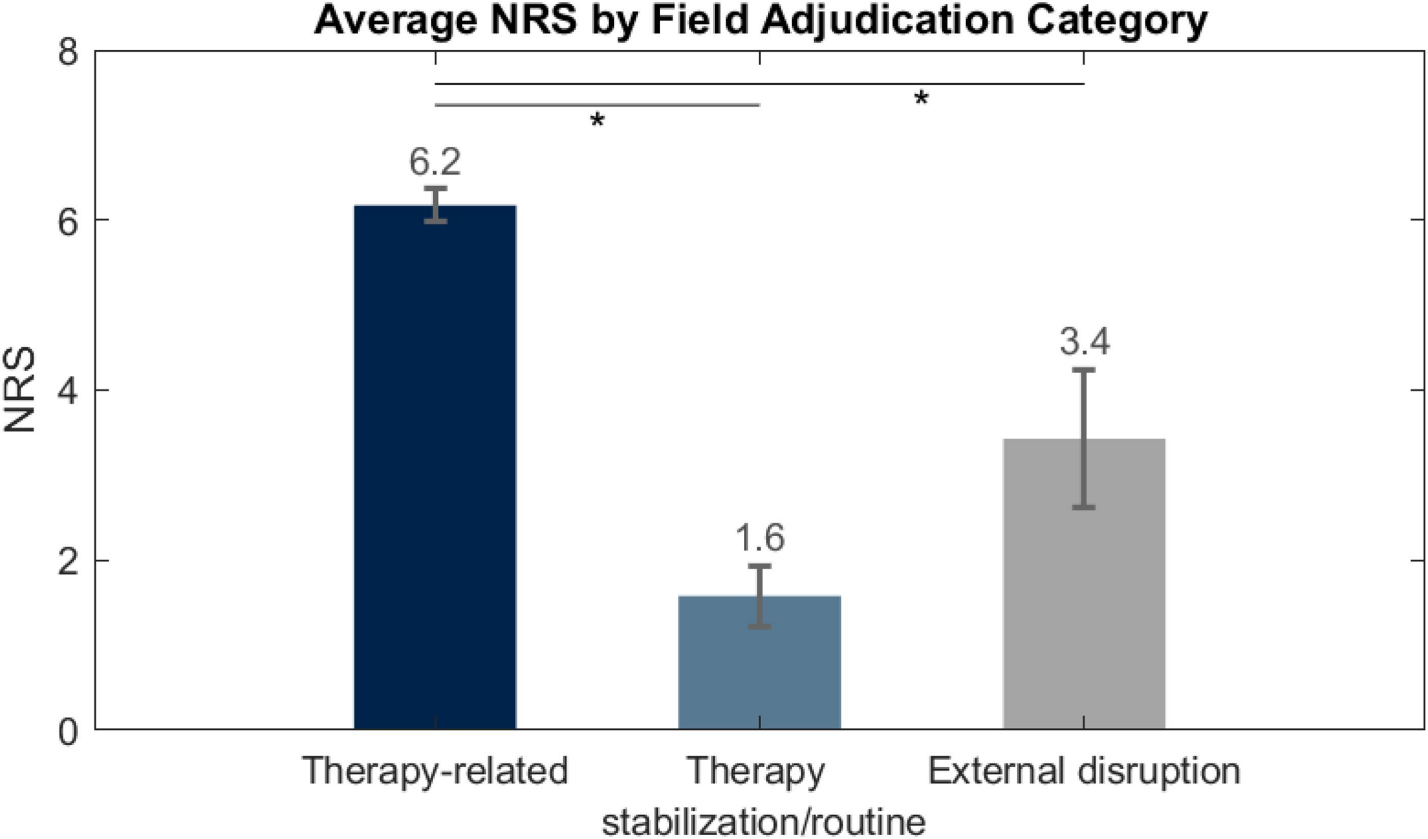
Pain intensity (NRS) at the time of outreach. Error bars show standard error. * indicates statistical significance *p* < 0.001 (linear fixed effects model).

Figure 4 summarizes pain intensity, measured using the numerical rating scale (NRS), at the time of outreach across adjudication categories. NRS completion rate was 93.4% from patients who responded (142/152). Patients classified as therapy-related reported significantly higher pain intensity at outreach than those classified as behaviorally meaningful stabilization/routine [linear fixed effects model, t(154) = −9.486, *p* < 0.0001] or externally driven disruption [linear fixed effects model, t(154) = −3.603, *p* = 0.0004], supporting the clinical face validity of the adjudication framework. Across categories for which NRS data were available, the overall pattern was consistent with the operational interpretation of the flags: cases judged to be therapy-related were associated with higher symptom burden at contact, whereas cases reflecting stabilization or routine adaptation tended to show lower reported pain.

These findings provide an additional layer of construct validity for the pilot workflow, showing that field-adjudicated therapy-related cases were not only operationally meaningful, but also associated with higher patient-reported pain at the time of outreach.

### 3.6 Intervention outcomes: downstream care actions following model flagging

After patient contact was established, ECT actions were determined according to the reported reason for therapy use patterns and the patient's current support needs. Intervention categories and operational descriptions are summarized in Table 4. These included therapy-related actions (reprogramming, therapy adjustment, and therapy discussion/goal realignment), escalation to clinician/sales representative/technical support, education, and monitoring without active intervention. The case results by patient care action are summarized in Table 5.

**Table 4:** Intervention outcomes and descriptions.

Intervention	Operational Description
Address therapy	Reprogramming: reprogram therapy parameters in a follow-up session
	Therapy adjustment: advise amplitude change or program switch
	Therapy discussion: discuss therapy options and/or realign patient goals
Escalation to clinician/rep/tech support	Advise patient to consult or contact clinician/tech support/sales rep on patients’ behalf
Education	Provide education to support therapy adherence
Monitor	Monitor without providing active support

**Table 5:** Intervention outcome summary by patient care actions.

Intervention	*n*	% of Reachable Cases (*N*_re_ = 152)
Address therapy	100	67.8%
Reprogramming	40	26.3%
Therapy adjustment	32	21.1%
Therapy discussion	31	20.4%
Escalation	29	19.1%
Escalation to clinician	19	12.5%
Escalation to rep/tech support	14	9.2%
Education	8	5.3%
Monitor	35	23.0%
All active support	117	77.0%

Categories are not mutually exclusive because a single case may result in more than one intervention, which results in % of reachable cases adding up to a value greater than 100%.

**Figure 5:**
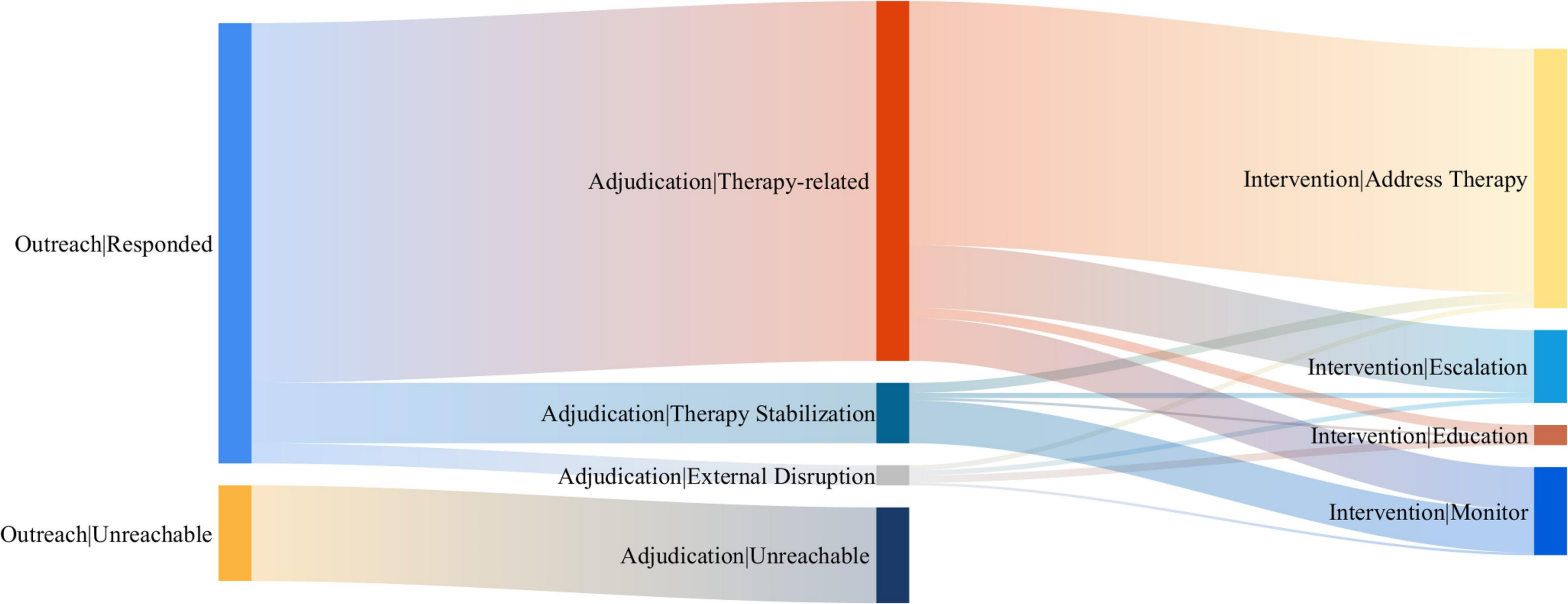
Sankey diagram showing case throughput from model flagging through ECT review, field adjudication, and downstream intervention outcomes.

Figure 5 illustrates the case throughput from model flagging to outreach, adjudication, and downstream intervention using a Sankey diagram. As expected, most therapy-related cases flowed toward therapy-related resolutions, including reprogramming, therapy adjustment without reprogramming, or therapy-focused discussion. In contrast, cases categorized as behaviorally meaningful stabilization/routine more often flowed toward monitoring or brief clarification. Cases driven by external disruptions were more likely to result in education, troubleshooting, or escalation to representative or technical support depending on the identified barrier.

This pattern supports the operational logic of the workflow: the model did not simply surface patients needing reprogramming, but rather provided meaningful enrichment for patients whose recent usage changes warranted some form of tailored supportive action. In practice, this included multiple actionable pathways beyond just reprogramming.

**Figure 6:**
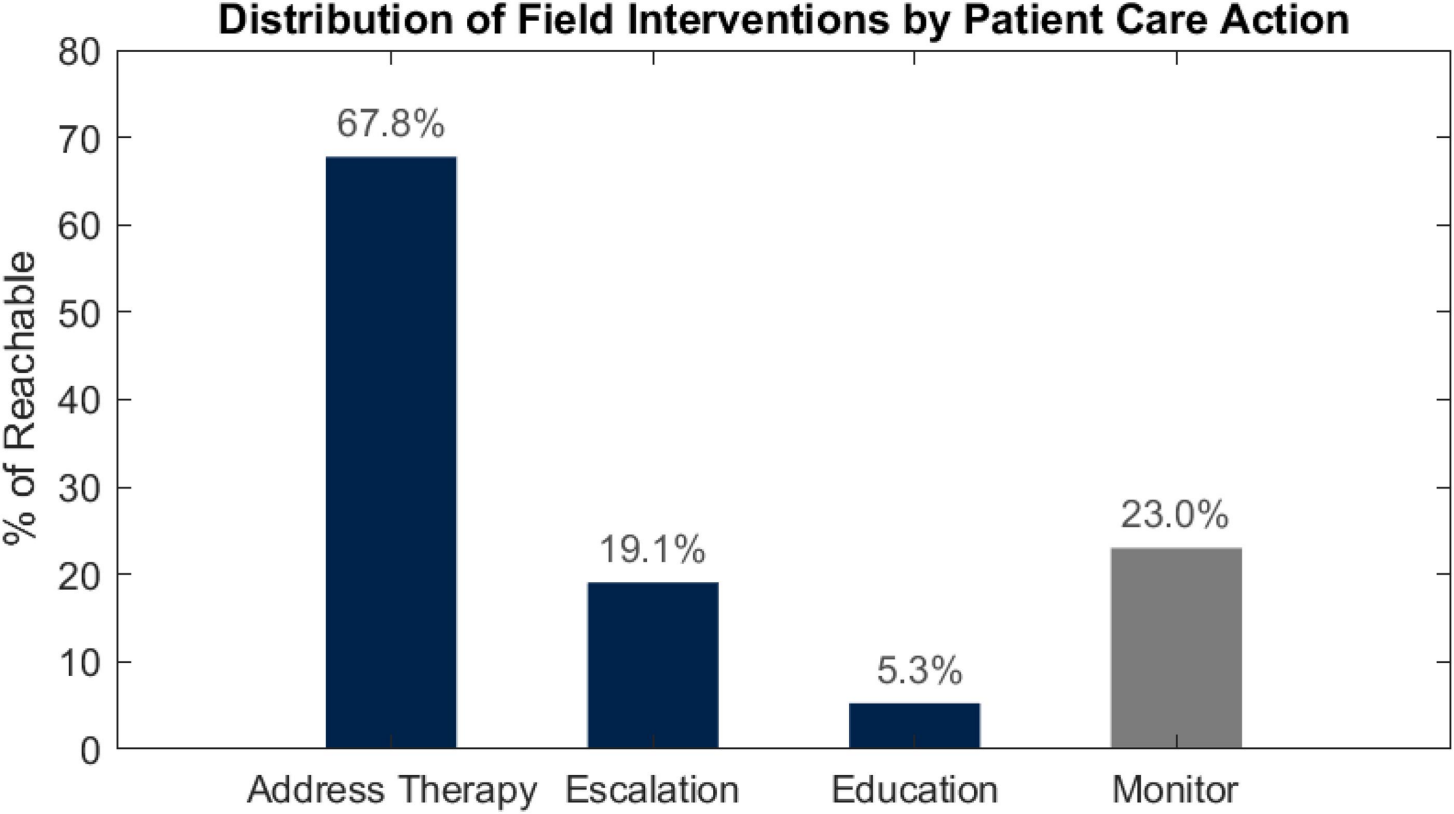
Distribution of field interventions by patient care action among reviewed cases. Field interventions represent patient care actions performed following ECT review, including addressing therapy, escalation, education, and monitoring. Predominance of addressing therapy indicates that reviewed cases frequently identified opportunities to provide additional support in response to patient needs. Categories are not mutually exclusive because a single case may result in more than one intervention.

Figure 6 shows the distribution of intervention outcomes. Because more than one intervention could result from a single reviewed case, intervention categories were not mutually exclusive. “Address therapy” actions constituted a substantial proportion of interventions at 67.8%—reprogramming (26.3%), therapy adjustment (21.1%), and therapy discussion (20.4%) (Table 5). In addition, escalation to clinicians occurred in 12.5% of cases, accounting for more than half of all escalations overall (19.1%). When including escalation and education, active support was provided in 77.0% of the cases, indicating that the practical value of the system extended beyond identifying candidates for reprogramming sessions. Some therapy-related cases were monitored without active support because a therapy-adjustment process had already been initiated before model flagging, while some non-therapy-related cases still benefited from education or clarification.

### 3.7 Pain score change after intervention

To descriptively summarize pain score change after outreach and subsequent care actions, percent NRS reduction (pNRSr) was calculated for 53 cases in which paired outreach and follow-up pain scores were available:

$$pNRSr=\frac{NRS_{outreach}-NRS_{\;followup}}{NRS_{outreach}}\times 100\text{\%}.$$

**Figure 7:**
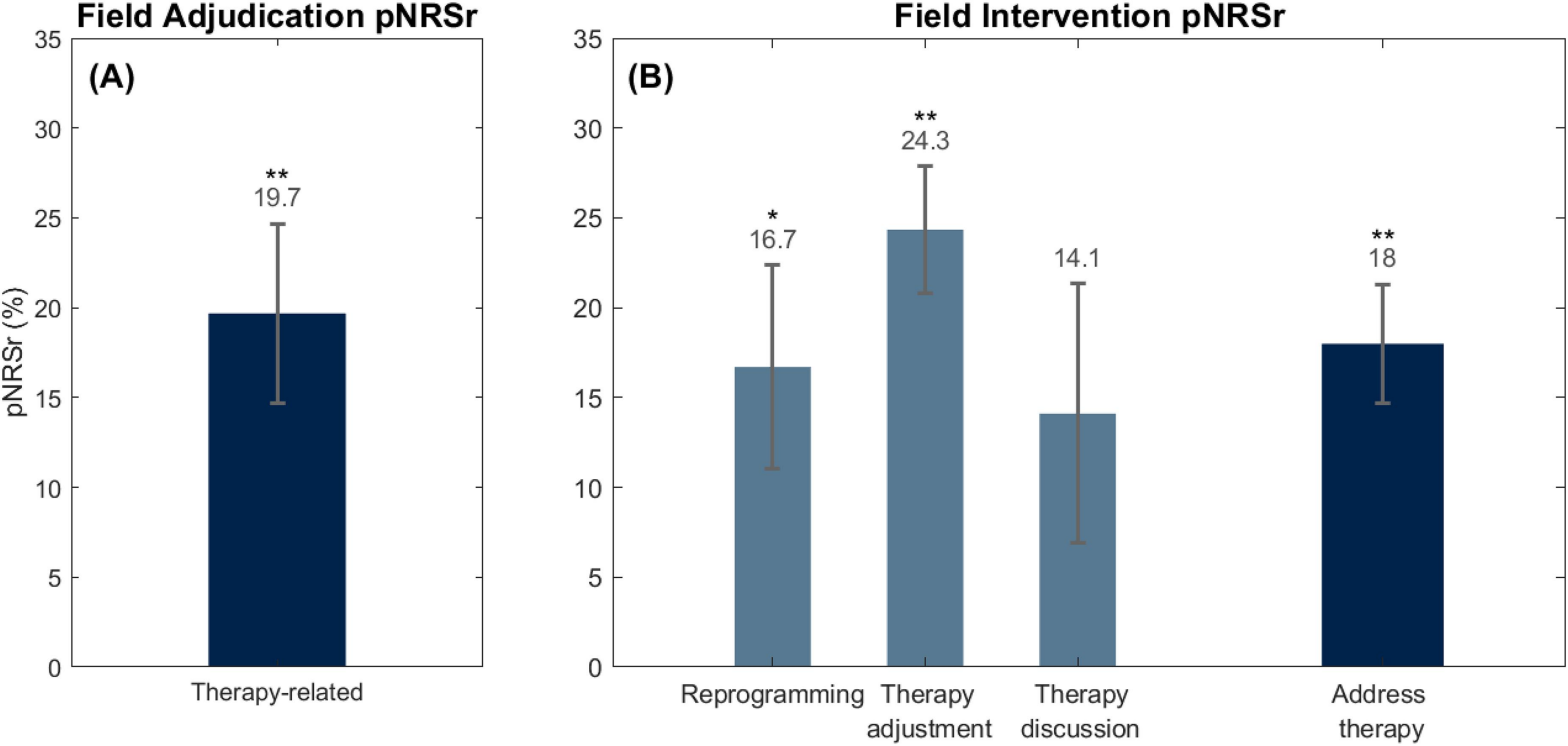
Percent NRS reduction (pNRSr) (**A**) by field adjudication category “Therapy-related” (left) and (**B**) by field intervention category “Address therapy” (far right) and its sub-categories “Reprogramming”, “Therapy adjustment”, and “Therapy discussion”. Error bars show standard errors. *, ** indicate one-tailed statistical significance of *p* < 0.05 and *p* < 0.01 from paired t-test against H0: pNRS ≤ 0, respectively.

Because follow-up pain scores were available only for a selective subset of reachable patients, this analysis should be interpreted as descriptive and hypothesis-generating rather than evidence of causal treatment effect. Figure 7 summarizes pNRSr by field adjudication grouping and by therapy-related intervention subtype.

When grouped by field adjudication, therapy-related cases showed a significant mean NRS reduction of 19.7% following outreach [paired t-test, t(52) = 3.936, one-tailed *p* = 0.0001], consistent with the intended use of the workflow to identify patients who might benefit from timely support. When grouped by intervention subtype, significant NRS reduction was observed not only after formal reprogramming, with a mean reduction of 16.7% [paired t-test, t(28) = 2.445, one-tailed *p* = 0.032, Bonferroni corrected], but also after therapy adjustment, which showed a mean reduction of 24.3% [paired t-test, t(11) = 4.210, one-tailed *p* = 0.0022, Bonferroni corrected]. Therapy discussion was also associated with a mean NRS reduction of 14.1%, although this did not reach statistical significance. When these intervention subgroups were considered together under the broader “Address therapy” category, the mean NRS reduction was 18.0% [paired t-test, t(50) = 3.774, one-tailed *p* = 0.0002].

This pattern is operationally relevant because observed post-outreach pain score decreases were not limited to cases involving technical reprogramming. Similar descriptive reductions were also observed after less extensive support actions, such as advising amplitude adjustment, program switching, or clarifying therapy expectations.

### 3.8 Representative cases

#### 3.8.1 Program-use behavior: seeking increased relief → reprogramming

**Figure 8:**
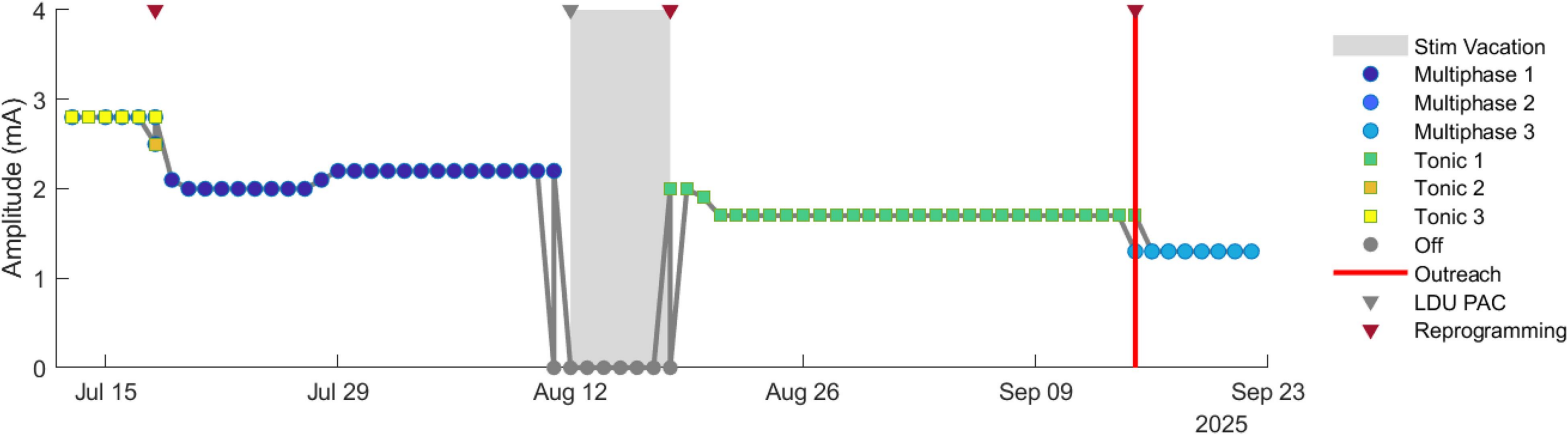
Program use behavior representing seeking increased relief → reprogramming.

Figure 8 illustrates one patient's 64-day program-use behavior and intervention history preceding the AI-informed outreach. During this period, the patient used a mixture of multiphase and tonic therapies along with multiple parameter settings in an effort to improve pain relief. The patient also underwent two reprogramming sessions. The later session was preceded by a stim holiday initiated during patient outreach for a low device usage (LDU) PAC trigger. Consistent with the model's feature-importance patterns (Supplementary), the combination of highly variable program-use behavior, including a sustained stimulus-off interval, together with prior follow-up event history, likely contributed to the case being flagged. During the Embrace AI-triggered outreach, the patient was advised to restart the optimization process and return to initial default settings. A reprogramming was performed on the same day, followed by another reprogramming the next month. The patient's pain score (NRS) was 7 at the time of outreach, decreased to 5 by the time of the second reprogramming, and ultimately improved to 3, which corresponds to pNRSr of 57.1%. The patient later reported high satisfaction after identifying a therapy setting that provided adequate pain relief.

#### 3.8.2 Program use behavior: new/worsened pain distribution → therapy adjustment

**Figure 9:**
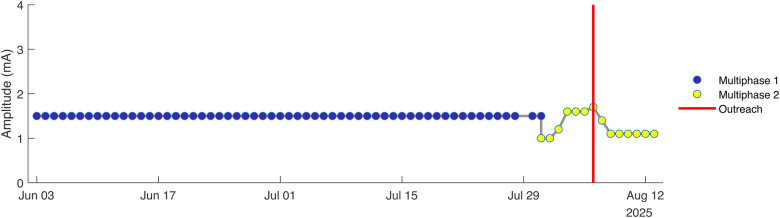
Program use behavior representing new/worsened pain distribution → therapy adjustment.

The patient shown in Figure 9 reported increased pain during a time period of competing health issues, which had prompted a switch to a different program. The patient reported leg cramping with the new program and was advised to reduce the amplitude to help alleviate the cramping. The patient reported a pain score of 8 at the time of outreach, which improved to 5 after therapy adjustment, resulting in pNRSr of 37.5%.

#### 3.8.3 Program use behavior: benefit stabilized → monitor

**Figure 10:**
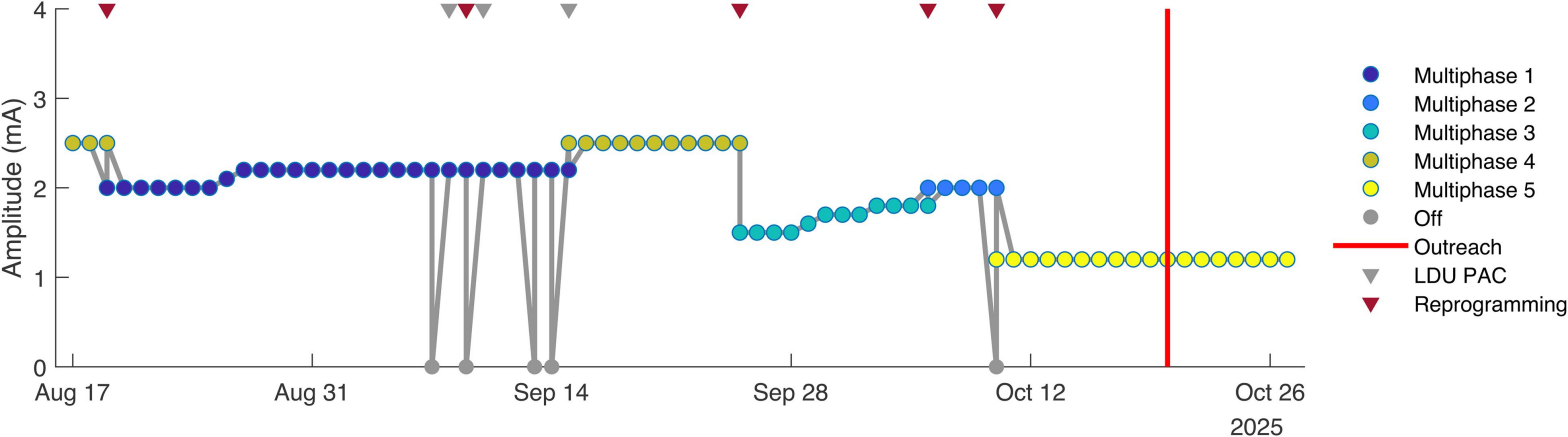
Program use behavior representing benefit stabilized → monitor.

The patient in Figure 10 underwent five reprogramming sessions and three LDU PAC triggers during the 64-day window preceding the AI-informed outreach as therapy optimization was ongoing. Following the last reprogramming session (Multiphase 5), therapeutic benefit stabilized. The patient reported a pain score of 2 and expressed great satisfaction with the latest therapy setting. Notably, this patient's program-use behavior and intervention history resembled the pattern observed from the patient in the first representative case who ultimately required reprogramming, including highly variable program use, multiple stimulus-off days (LDU triggers), and repeated reprogramming sessions. However, field adjudication indicated a different pain experience context in this case, showing that similar observable behavior patterns may arise from different underlying reasons. Together, these cases illustrate the heterogeneity of patient behavior and highlight the importance of human adjudication in interpreting model-flagged cases.

## 4 Discussion

This prospective real-world pilot evaluated Proactive Intelligence as a workflow-integrated, human-in-the-loop recommender designed to support post-implant SCS care under routine operational constraints. Rather than treating the model as a standalone classifier, this study evaluated whether model-prioritized behavioral signals could be translated into useful field actions by a support team to improve therapeutic efficacy for patients in need. The findings suggest that the deployed workflow provided enrichment for patients with therapy-related or otherwise meaningful behavioral changes. It enabled targeted outreach and supported multiple downstream actions including reprogramming, therapy adjustment/discussion, education, escalation, and monitoring. In this setting, the value of the system was not defined solely by algorithmic discrimination, but by the extent to which model outputs could be converted into timely and actionable support within a real-world care process.

The value proposition for the Proactive Intelligence system is consistent with a broader shift in neuromodulation interventions towards remote, longitudinal care. Prior consensus guidance has emphasized that remote monitoring and remote programming may help address the limitations of in-person-only follow-up and support temporal enhancement of individualized care (8). This pilot aligns with these goals and extends state-of-the-art remote technologies by adding a model-assisted prioritization layer to address an anticipated surveillance gap: even when remote device data are available, meaningful changes in patient state may occur between scheduled touchpoints and may not be surfaced quickly enough to support timely action. This results in inefficient workflows leading to suboptimal impact. This real-world pilot evaluates a workflow-integrated AI system that uses automatically collected patient-device interaction data, without requiring additional patient-entered surveys, wearable devices, or other active data-collection tasks, to prioritize patients for targeted outreach in post-implant SCS care. More broadly, digital-health frameworks in neuromodulation have emphasized that connected devices, remote data streams, and predictive analytics are most valuable when embedded into patient-centered care pathways rather than treated as standalone analytic endpoints (15).

An important finding of this pilot is that many flagged cases were operationally meaningful even when they did not lead to therapy reprogramming. In a conventional supervised-learning framework, such cases might be classified as false positives. However, in this positive-unlabeled setting, the absence of a documented reprogramming event does not establish absence of need. Field adjudication showed that many reviewed patients had therapy-related issues, while others reflected meaningful stabilization, intentional routine changes, or externally driven disruptions. This distinction is important because it reframes deployment performance as the output of a sociotechnical workflow rather than of a binary classifier alone. In other words, the model surfaced candidates for review while ECT interaction and patient context determined further downstream care actions such as therapy-related support, education, escalation, or continued monitoring.

Among patients with follow-up NRS data, observed pain reductions were not limited to technical reprogramming but also associated with therapy adjustment and discussion. Operationally, this pattern suggests that proactive outreach may create value through multiple channels, including reinforcing therapy use, clarifying expectations, advising program or amplitude changes, and identifying barriers before they escalate into more resource-intensive problems. The escalation pathway was also an important feature of the workflow. By enabling escalation to clinicians, field representatives, and technical support, the system linked early behavioral signal detection to a downstream care structure capable of responding at different levels of need. In practice, some patients required formal clinical review, coordination, or escalation even when they did not proceed directly to reprogramming. The availability of this pathway therefore strengthened the practical relevance of the workflow, because predictive signals are most useful when paired with a clear route to action.

These findings underscore the importance of contextual interpretation. The model had access only to structured usage features, operational variables, and limited demographics. It did not directly observe interim clinical events, psychosocial stressors, life disruptions, or patient narratives that may shape device use. That the system still identified and enriched meaningful cases under these constraints is encouraging, but it also reinforces why human review remains necessary. The model surfaced candidates for review, whereas interpretation and action required contextualization by the ECT. In our setting, this contextual layer was essential because superficially similar objective patterns could reflect very different underlying circumstances, including ongoing optimization, usability barriers, competing health issues, or waning therapy benefit. Patient history and ECT adjudication were therefore not merely supplementary but were necessary for converting model outputs into operationally appropriate actions.

Geographic region was among the top model features, which further supports the need for contextual interpretation. This variable likely reflects contextual and operational influences on therapy-use behavior in addition to a direct patient-level signal. Patient behavior is shaped in part by the education, instruction, and follow-up support provided during routine care, and those factors may vary across settings. Qualitative work in SCS has shown that patients have substantial educational and informational needs related to device use and the broader care journey, supporting the idea that therapy behavior may be influenced by how education and support are delivered in practice (16). Geographical region may therefore act as a proxy for variation in the surrounding care environment, including local practice patterns and indirect operational inputs communicated through field teams. This interpretation is important because it suggests that longitudinal usage behavior is shaped not only by patient need, but also by how therapy usage is taught, reinforced, and supported in practice.

To clarify the positioning of this pilot relative to adjacent digital health and clinical AI literatures, Table 6 summarizes the typical data sources and implementation focus of related work. The table is intended to contextualize the present implementation study rather than to provide a systematic review or performance benchmark. The present pilot intersects with remote SCS management, digital biomarker and digital phenotyping research, clinical sequence modeling, and positive-unlabeled clinical learning, but differs in its implementation focus and data context, specifically the use of passively collected SCS device-interaction behavior within a human-in-the-loop care workflow. Clinical sequence and early-warning models commonly use EHR time series, vital signs, laboratory values, monitoring streams, or clinical notes to predict deterioration or other clinical events (17). In contrast, Proactive Intelligence used passively collected SCS therapy-use and device-interaction behavior to prioritize review within an existing remote care workflow, rather than to serve as a general-purpose early-warning benchmark model. Positive-unlabeled clinical learning is also relevant because documented therapy-adjustment events represent high-confidence positives, whereas patient-days without documented therapy adjustment are unlabeled rather than confirmed negative (18, 19). However, the present study did not develop a new positive-unlabeled learning method but rather, the positive-unlabeled framing was used to interpret label scarcity and the limitations of conventional false-positive/false-negative interpretation in real-world post-implant care.

**Table 6:** AI-augmented remote SCS management relative to adjacent literature.

Literature area	Typical data source	Implementation focus
Remote SCS management (7, 15)	Remote monitoring, remote programming, device status	Support longitudinal SCS therapy management and patient-centered remote care
Clinical sequence models/early warning models (17)	EHR time-series, vital signs, labs, monitoring streams, clinical notes	Predicts deterioration or other clinical events from longitudinal clinical data
Positive-unlabeled clinical learning models (18, 19)	Labeled positive cases with unlabeled or unreliable negative cases, often from EHR or administrative data	Addresses incomplete labels where true negatives are difficult to define
Digital biomarkers/phenotyping in pain (20–22)	Wearables, smartphone sensors, physiological/activity data, patient-reported data	Characterizes pain, function, behavior, or disease state using passive or semi-passive signals
Present study	Passive SCS device interaction behavior, patient/care history/context features	Prioritizes patient care review within AI-augmented remote SCS management

The distinction from digital biomarker and digital phenotyping work is also important. Prior work has shown both the promise and the limitations of wearable-derived and longitudinal behavioral data, while also elucidating a key distinction of the present pilot. Outside neuromodulation, wearable-based machine learning has been used to support individualized prediction from dense longitudinal data streams. However, these efforts have not evaluated a fully workflow-integrated patient care pathway in which passively collected device-interaction data are translated into patient outreach for potential therapy management support. Nan et al. developed personalized ML models to predict wellbeing and empathy from smartwatch and ecological momentary assessment data (20), while Chiang et al. used wearable and behavioral data to generate personalized lifestyle recommendations for blood-pressure improvement (21). In SCS, Patterson et al. showed that passive biomarkers such as heart rate, heart-rate variability, step count, and stand time can correlate with pain and broader therapy outcomes (22). However, that study also noted that objective signals are influenced by multiple confounding factors and do not fully capture the lived context surrounding a patient's therapy course (22). In operational deployment, that missing context becomes critical. Additional information from patient history, prior follow-up events, stimulus-off behavior, and adjudicated reasons for nonresponse or stabilization are extremely important for determining whether a flag reflects a need for therapeutic intervention, goal realignment, education, troubleshooting, or continued observation. In that sense, the remote-care paradigm supported by this pilot is best understood as sociotechnical: objective digital signals enable more efficient surveillance, but contextual behavioral information and human adjudication are what make those signals actionable.

The reviewed cohort also differed from the overall and model-flagged cohorts by primary indication. Most notably, CRPS was overrepresented among reviewed patients relative to both the overall and model-flagged cohorts, while degenerative disk disease was also modestly enriched. These differences may not reflect model behavior alone. CRPS is a complex pain condition often requiring multidisciplinary, longitudinal support including repeated assessment, patient education, rehabilitation, and escalation to specialized care when needed (23, 24). Furthermore, it is recommended that CRPS care should be matched to disease severity and complexity (23) and that assessment should be repeated over time because both disease course and treatment effects may change the clinical picture (24). Therefore, the enrichment of CRPS among reviewed cases may indicate that these patients generate more persistent or operationally visible behavioral shifts, making them more likely to survive ranking and enter review. In this context, Proactive Intelligence may help the ECT identify patients who may require more support and route them toward earlier outreach. This observation is hypothesis-generating and supports future evaluation of how model detection, prioritization logic, and indication-specific care patterns jointly shape workflow yield.

Several limitations should be considered. First, this was not a randomized study and did not include a concurrent control arm comparing Proactive Intelligence with standard care. Therefore, causal inference regarding patient benefit is limited, and the study cannot establish superiority over standard care. The pilot was designed as an implementation and feasibility evaluation, with emphasis on enrichment, actionability, and workflow performance rather than comparative efficacy. Proactive Intelligence should therefore be interpreted as an incremental AI-supported surveillance layer added to existing care pathways, not as a replacement for or proven superior alternative to standard care.

Second, several model development limitations should be noted. The model development process did not include a separate validation set, formal hyperparameter search, cross-validation, calibration analysis, systematic comparison with alternative architectures, or formal ablation analyses. Fixed prespecified parameters were used, and the architecture was selected as a pragmatic multimodal structure for prospective workflow deployment rather than through formal model-selection experiments. Therefore, the reported AUC-ROC should be interpreted as a development-stage diagnostic under the study's label structure, not as evidence that the model is superior to simpler aggregate-feature models or alternative temporal architectures.

Operational variables also introduce important interpretive limitations. Follow-up history and geographical region may encode care process context, including local practice patterns, documentation processes, patient education, field team interaction, and access to support. Because positive labels were derived from documented care events, these variables may introduce care process learning or target-leakage risk and should not be interpreted as intervention-need features. Because feature removal experiments were not performed, the incremental contribution and potential leakage effect of the time-series branch, demographic variables, follow-up history, and geographical region cannot be determined from the present study. Future model development work should include ablation analyses excluding operational variables to evaluate whether model performance and operational yield are preserved when care process features are removed.

Third, external generalizability is limited by the single operational setting, single device ecosystem, and fixed ECT workflow evaluated in this pilot. The observed enrichment, actionability, and review yield may depend on the data environment, the structure of the ECT, local escalation pathways, patient mix, and existing follow-up practices. Accordingly, these findings should be interpreted as context-specific evidence of feasibility and actionability within this workflow, rather than as evidence that the same model, threshold, or workflow would perform similarly in other settings.

Fourth, field adjudication was designed to characterize the care context underlying AI-prioritized device interaction patterns rather than to assign binary model-correctness labels. Accordingly, the prospective adjudication workflow did not include a separate “no contextual finding” or model-failure category. Cases in which outreach did not identify a therapy-related concern could therefore overlap with therapy stabilization/routine or other low-actionability contexts. We do not interpret these cases as conventional false positives because no pre-existing ground truth was available. At the same time, we also do not include therapy stabilization/routine cases in the primary actionable outcome.

Fifth, outcome analyses involving pain scores were subject to data gaps and likely selection bias, and limitations of the sing-arm observational design. Follow-up NRS data were available only for a subset of patients who responded and provided evaluable follow-up information. Patients with available follow-up pain scores may differ systematically from those without follow-up data, including symptom severity, engagement with outreach, perceived benefit, or likelihood of ongoing therapy optimization. Regression to the mean may also have contributed to the observed reductions, particularly if patients were reviewed during periods of worsening pain, changing therapy-use behavior, or increased likelihood of care engagement. Therefore, observed NRS reductions should be interpreted as descriptive and hypothesis-generating rather than as definitive evidence of treatment effect or as representative of all reviewed patients. Future controlled studies are needed to determine whether AI-prioritized outreach improves pain outcomes compared with standard care alone.

Sixth, the reported field performance reflects the care ecosystem rather than the model alone. Model outputs were filtered through post-prediction prioritization and then constrained in capacity by study design, such that observed outcomes represent the combined effect of model behavior, ranking logic, and operational workflow. The model's low sensitivity should also be interpreted in this context. In the temporally held-out set used during model development, the model achieved sensitivity of 8.6% at the selected operating threshold, indicating that only a minority of positive-labeled events were captured. This reflected the intended high-specificity design of the workflow, which prioritized enrichment and precision over comprehensive detection. Proactive Intelligence should therefore be interpreted as an incremental prioritization layer added to existing care pathways, not as a standalone surveillance system or replacement for standard follow-up. Patients retained access to existing care pathways, including patient-initiated contact, routine follow-up, remote monitoring review, and clinician-directed escalation. Thus, the primary implication of low sensitivity is that some patients may not receive the incremental AI-supported outreach rather than that an existing care pathway was removed. At the same time, SHAP feature-importance patterns in the reviewed-case dataset were highly similar to those observed in the training dataset, suggesting that the prioritization process did not distort the underlying feature signal or introduce obvious systematic shift in the types of cases ultimately reviewed. Nonetheless, some selection effects related to operational capacity and downstream workflow cannot be excluded.

Finally, the development labels captured only documented reprogramming-related events and therefore likely underrepresented other forms of clinically meaningful need Although biweekly retraining was performed to mitigate potential real-world data drift, post-retraining monitoring in this pilot was limited to randomly split test AUC-ROC. Randomly split test AUC-ROC remained consistently around 0.99, indicating that each retrained model continued to capture the positive-labeled input patterns. However, this metric should be interpreted as a quality-control measure rather than evidence of stable out-of-sample performance. Future improvements should include prospective monitoring of model stability after each retraining cycle using temporally separated holdout data, along with prospective analysis of missed-event patterns to determine whether false negatives cluster by therapy-use behavior, geography, time since last follow-up, indication, or other patient characteristics.

Despite these limitations, this pilot provides a practical demonstration of how machine learning can be integrated into post-implant SCS support in a manner that is operationally useful and clinically conservative. More broadly, the findings suggest that the value of ML in longitudinal remote-care settings may lie less in autonomous prediction of narrowly defined endpoints and more in surfacing behavior changes that enable timely human-mediated intervention. Future work should evaluate this approach in broader operational settings, assess subgroup-specific yield across indications, and further refine the prioritization and escalation pathways that connect model signals to patient care.

## 5 Conclusion

This prospective real-world pilot showed that Proactive Intelligence can be operationally integrated into post-implant SCS support as a human-in-the-loop recommender that helps prioritize patients for follow-up to optimize clinical workflows. To our knowledge, this is the first real-world evaluation of a patient care workflow that uses passively collected SCS data to enable targeted outreach without adding data-entry burden on patients. The model enriched workflow identified meaningful behavioral changes and supported a range of downstream actions beyond reprogramming alone, including therapy adjustment, discussion, education, escalation, and monitoring. These findings suggest that the value of ML in longitudinal SCS care may lie less in autonomous endpoint prediction and more in surfacing patients who may benefit from timely human review. Future work should evaluate this approach on a larger scale and further refine the pathways that connect model-identified behavioral signals to patient benefit.

## Data Availability

The datasets presented in this article are not readily available because the patient-level data analyzed in this study are subject to the following restrictions: the data contain PHI, device interaction records, care team documentation, and operational workflow data that may include protected health information or potentially reidentifiable information from study participants and therefore cannot be made publicly available. Requests for additional information or limited access to de-identified or aggregated data may be submitted and will be evaluated on a case-by-case basis, subject to applicable privacy requirements, patient consent, institutional approval, and data-use agreements. Requests to access the datasets should be directed to Neuro.Clinical.Technology@biotronik.com.
