# Supplementary material for: From behavioral signals to therapy management: a real-world pilot implementation of human-in-the-loop AI workflow in proactive patient care
Source: Front Digit Health. 2026 Jul 27;8:1887889. doi: 10.3389/fdgth.2026.1887889 (PMC13454061; doi:10.3389/fdgth.2026.1887889)
Supplement: Supplementary file 1 [file Datasheet1.docx]

Supplementary Material

# 1 Supplementary Data

Model interpretability was assessed using SHAP (SHapley Additive exPlanations) (14) values computed with a Kernel SHAP approach on the training dataset (Supp Fig. 1), temporally held-out dataset (Supp Fig. 2), and reviewed-case dataset (Supp Fig. 3). Global feature importance was summarized using mean absolute SHAP values.

Feature importance rankings were broadly consistent across the training and temporally held-out datasets, with substantial overlap also observed in the reviewed-case dataset. This pattern suggests that the model relied on stable behavioral and device-interaction signals, and that similar feature families remained influential in the subset of cases that progressed to operational review.

Because many inputs were temporal or aggregated features, signed SHAP values were less straightforward to interpret at the individual-feature level. Accordingly, interpretation focused on global importance and cross-dataset consistency rather than directional attribution. SHAP values do not imply causality, but they support that the model’s predictions were driven by coherent and clinically relevant patterns in longitudinal therapy usage data.

Supplementary Figure 1. Feature importance computed on training dataset.

Supplementary Figure 2. Feature importance computed on temporally held out test dataset.

Supplementary Figure 3. Feature importance computed on reviewed-case dataset
